# Gut microbes in cardiovascular diseases and their potential therapeutic applications

**DOI:** 10.1007/s13238-020-00785-9

**Published:** 2020-09-28

**Authors:** Ling Jin, Xiaoming Shi, Jing Yang, Yangyu Zhao, Lixiang Xue, Li Xu, Jun Cai

**Affiliations:** 1 Center of Basic Medical Research, Institute of Medical Innovation and Research, Peking University Third Hospital, Beijing, 100191 China; 2 Department of Obstetrics and Gynecology, Peking University Third Hospital, Beijing, 100191 China; 3 Heart Center and Beijing Key Laboratory of Hypertension, Beijing Chaoyang Hospital, Capital Medical University, Beijing, 100043 China; 4 Hypertension center of Fuwai Hospital, State Key Laboratory of Cardiovascular Disease, National Center for Cardiovascular Diseases, Chinese Academy of Medical Sciences and Peking Union Medical College, Beijing, 100037 China

**Keywords:** gut microbiota, cardiovascular diseases, action mechanism, therapeutic applications

## Abstract

Microbial ecosystem comprises a complex community in which bacteria interact with each other. The potential roles of the intestinal microbiome play in human health have gained considerable attention. The imbalance of gut microbial community has been looked to multiple chronic diseases. Cardiovascular diseases (CVDs) are leading causes of morbidity worldwide and are influenced by genetic and environmental factors. Recent advances have provided scientific evidence that CVD may also be attributed to gut microbiome. In this review, we highlight the complex interplay between microbes, their metabolites, and the potential influence on the generation and development of CVDs. The therapeutic potential of using intestinal microbiomes to treat CVD is also discussed. It is quite possible that gut microbes may be used for clinical treatments of CVD in the near future.

## Introduction

Cardiovascular diseases (CVDs) have taken center stage as the leading causes of morbidity and mortality worldwide. The cause of CVD is still under investigation, but thought to be influenced by genetic and environmental factors.

Recent studies have shed light on the potential roles of the intestinal microbiome in CVD. Microbes in the intestine vastly outnumber the total number of human cells. They form a complex community of interacting organisms and are even collectively regarded as a human organ (Clemente et al., 2012). Starting at childbirth, many factors influence the composition, metabolism and functions of this microbial community. Many studies have indicated that gut microbiome imbalance may play a role altering CVDs susceptibility by influencing aspects of the immune response, obesity, insulin resistance, atherosclerosis and thrombosis susceptibility factors. The microbita may also communicate with distal host organs through their metabolites (Tang and Hazen, 2017).

In this review, we summarize the complex interplay between microbes (and their metabolites) and their potential roles in the generation and development of CVDs. Moreover, possible therapeutic strategies for modulating the intestinal microbes to alter the generation and development of CVDs are discussed.

## Microbiota dysbiosis and cvds

Microbiota dysbiosis refers to the imbalance in host-microbial interactions. Changes in either microbial composition or community-derived factors (such as metabolites or genotoxins) can be directly or indirectly associated with enhanced disease susceptibility. These microbiome based factors may cause activation of signaling pathways and ultimately lead to the pathophysiological diseases of CVDs. Moreover, these components have the potential to be targeted for therapeutic applications.

### Changes in gut microbial composition

The gut microbiota consists of four major phyla: *Firmicutes*, *Bacteroidetes*, *Actinobacteria* and *Proteobacteria*. *Bacteroidetes* and *Firmicutes* together account for the majority of the gut community in healthy adults, and the *Firmicutes*/*Bacteroidetes* ratio is viewed as a health indicator of intestinal microbiota (Qin et al., 2010). However, microbial composition differs between individuals and is dynamically sensitive to host factors as well as environment parameters. Interestingly, both increased abundance of host opportunistic pathogens (e.g., *Escherichia coli*, *Clostridium ramosum*, *Bacteroides caccae*, and *Eggerthella lenta*) and reduced abundance of short-chain fatty acid (SCFA) producing bacteria (e.g., *Roseburia*, *Faecalibacterium* and *Eubacterium rectale*) have been associated with increased risks for developing CVDs (Gozd-Barszczewska et al., 2017; Yan et al., 2017). Therefore, both the types of the microorganisms and their relative abundance are factors that may alter the sensitivity of developing CVDs (Franzosa et al., 2015).

Studies of microorganisms have been revolutionized since the introduction of bacterial genome analyses. Two sequencing methods, 16S rDNA sequencing and metagenomic sequencing, have commonly been used for assessment of microbial compositions and their relative abundance. 16S sequencing detects differences in the hypervariable regions in bacterial genomes by targeting conserved regions that surround the variable regions. The main drawback of this technique is that it is insufficient for species-level resolution since their differences are often minor. On the contrary, shotgun metagenomics detect the entire genomes of all organisms at present, leading to increased sensitivity allowing the detection of both known and unknown microorganisms. In this part, we list changes of gut microbial composition associted with CVDs (Table 1).

**Table 1:** Altered gut microbial compositions associated with CVDs

Species	Technique	Illness-associated changes in gut microbial abundance		References
		Decrease	Increase	
Atherosclerosis and coronary artery disease				
Human	Metagenomic sequencing	*Bacteroides* and *Prevotella*	*Streptococcus* and *Escherichia*	(Jie et al., 2017)
Human	Terminal restriction fragment length polymorphism.	*Bacteroides* and *Prevotella*	Order *Lactobacillales*	(Emoto et al., 2016)
Human	Metagenomic sequencing	*Roseburia* and *Eubacterium*	*Collinsella*	(Karlsson et al., 2012)
Human	16S sequencing	*Clostridium*, *Faecalibacterium*	*Prevotella*	(Gozd-Barszczewska et al., 2017)
Mice	16S sequencing	*Roseburia*		(Kasahara et al., 2018)
Human	16S sequencing	*Burkholderia*, *Corynebacterium* and *Sediminibacterium*, unclassified *Comamonadaceae*, *Oxalobacteraceae*, *Rhodospirillaceae*, *Bradyrhizobiaceae* and *Burkholderiaceae*	*Curvibacter*, unclassified *Burkholderiales*, *Propionibacterium*, *Ralstonia*	(Ziganshina et al., 2016)
Hypertension				
Rat	16S sequencing	Family *Veillonellaceae*	Plasma acetate and heptanoate	(Mell et al., 2015)
Human	Metagenomic sequencing		*Prevotella* and *Klebsiella*	(Li et al., 2017a)
Human	Metagenomic sequencing	*Roseburia* spp., *Faecalibacterium prausnitzii*,	*Klebsiella* spp., *Streptococcus* spp., and *Parabacteroides merdae*	(Yan et al., 2017)
Human	16S sequencing	Butyrate-producing bacteria *Odoribacter*		(Gomez-Arango et al., 2016)
Heart failure				
Human	16S sequencing	*Blautia*, *Collinsella*, *uncl*. *Erysipelotrichaceae* and *uncl*. *Ruminococcaceae*		(Luedde et al., 2017)
Human	Incubation with a selective agar		*Campylobacter*, *Shigella*, *Salmonella*, *Yersinia Enterocolitica*,	(Pasini et al., 2016)
Human	16S sequencing	*Faecalibacterium*	*Lactobacillus*	(Kamo et al., 2017)
Human	Metagenomic sequencing	*Faecalibacterium prausnitzii*	*Ruminococcus gnavus*	(Cui et al., 2018)
Atrial fibrillation				
Human	Metagenomic sequencing	*Faecalibacterium*, *Alistipes*, *Oscillibacter*, and *Bilophila*	*Ruminococcus*, *Streptococcus*, and *Enterococcus*,	(Zuo et al., 2019a)

#### Atherosclerosis and coronary artery disease

Atherosclerosis is a chronic inflammatory disease characterized by the dysfunction of vascular cells and the accumulation of low-density lipoprotein particles in plaques (Davignon and Ganz, 2004; Libby et al., 2002). *Staphylococcus* species, *Proteus vulgaris*, *Klebsiella pneumoniae* and *Streptococcus* species have been identified in both of the atherosclerotic lesions and the gut of the same individual, which suggest the involvement of gut microbiota (GM) in atherosclerosis development (Ott et al., 2006). Certain types of gut bacteria have been identified as novel contributors to the progression of atherosclerosis. Middle-aged men in eastern Poland with improper total cholesterol levels and LDL-C values are rich in *Prevotella,* low of *Clostridium* and *Faecalibacterium* levels (Gozd-Barszczewska et al., 2017), while Chinese atherosclerotic cardiovascular disease patients show a relative reduction in *Bacteroides* and *Prevotella*, and enrichment in *Streptococcus* and *Escherichia* (Jie et al., 2017). The opportunistic pathogenic genus *Collinsella* has been found enhanced in patients with symptomatic atherosclerosis. Intriguingly, some microbial species reduce the risks of atherosclerotic plaque formation. For example, the relative depletion of butyrate-producing bacterial genus of *Roseburi*a and *Eubacterium* have been found to be inversely correlated with atherosclerotic lesion development in patients and genetical mouse models (Karlsson et al., 2012; Kasahara et al., 2018). It is speculated that these microorganisms may influence the inflammatory status of the host. Moreover, colonization by diverse bacteria rather than a single pathogen can impact plaque formation and stability. The bacterial community in atherosclerotic plaque samples has been found to be correlated with clinical parameters such as total cholesterol, alanine aminotransferase and fibrinogen levels (Ziganshina et al., 2016). However, it is unclear which species play a leading role in the contributing CVDs. The mechanism of how the microbiota influences the atherosclerosis development is of great interest of future investigation (Jin et al., 2019).

#### Hypertension

High blood pressure is a prevalent cause of CVDs worldwide. The first evidence suggesting the involvement of gut microbiota in hypertension pathogenesis was observed in rats on the effect of antibiotic treatment on blood pressure (Honour et al., 1985). Subsequently, reduced levels of microbial richness, biodiversity and evenness have been identified in the fecal microbiota of hypertensive animal models and human patients. In addition, the severity of hypertension was found to be associated with the numbers of hypertension related bacterial species, increased *Firmicutes*/*Bacteroidetes* ratios, greater abundances of opportunistic pathogenic taxa (e.g., *Klebsiella* spp., *Streptococcus* spp.), and reduced populations of acetate-/butyrate-producing bacteria (Mell et al., 2015; Yan et al., 2017; Yang et al., 2015). It has been reported that microbial characteristics are similar between prehypertensive and hypertensive populations, with obvious overgrowth of *Prevotella* and *Klebsiella* bacteria in both groups (Li et al., 2017a). The abundance of butyrate producing genus *Odoribacter* and butyrate production are inversely associated with blood pressure levels in women with higher risk of developing pregnancy-induced hypertension and preeclampsia (Gomez-Arango et al., 2016).

Hypertensive gut microbiome exhibits increased membrane transport, lipopolysaccharide biosynthesis and steroid degradation. Dysbiosis of the microbiota might be partially mediated byvitamin D3 deficiency (Zuo et al., 2019c). Numerous studies have investigated the relationship between hypertension and GM in humans and in rat animal models (Adnan et al., 2017; Durgan et al., 2016; Li et al., 2017a; Mell et al., 2015). Santisteban et al. find that alterations in blood pressure relevant microbial communities may lead to hypertensive gut pathological changes (Santisteban et al., 2017). Results from these studies have associated the gut microbiota with the development of hypertension although the mechanisms of how they function remain elusive.

#### *Heart failure* (*HF*)

HF, a disease characterized by a reduced ability of the heart to pump enough blood and oxygen to meet the body’s needs, is often the end clinical stage of many CVDs. Altered microbial composition has been reported in patients with HF (Luedde et al., 2017). HF-related multisystem disorder often display impaired intestinal barrier functions, this may lead to enhanced interaction between the intestinal microtiota with the host intestinal mucosa. Consequently, this may result in increased circulating lipopolysaccharides levels and the activation of downstream inflammatory responses (Sandek et al., 2007; Sandek et al., 2012). Furthermore, patients with comorbid HF are reported to experience *Clostridium difficile* infection (CDI) more frequently (Mamic et al., 2016).

Sequencing studies on HF cohorts consistently found reduced microbial diversity and depletion of several butyrate producing microorganisms. Enhanced populations of pathogenic *Campylobacter*, *Shigella* and *Salmonella* bacteria as well as less abundant *Eubacterium rectale* and *Dorea longicatena* bacteria were isolated from fecal samples of HF patients (Kamo et al., 2017; Pasini et al., 2016). In addition, certain HF patients with highly reduced left ventricular ejection fractions showed significant reductions in *Blautia*, *Collinsella*, unidentified *Erysipelotrichaceae* and *Ruminococcaceae* spp. populations (Luedde et al., 2017). Chronic HF (CHF) patients demonstrated reduced *Faecalibacterium prausnitzii* and increased *Ruminococcus gnavus* populations (Cui et al., 2018). The microbiota also varies according to age. Compared with younger patients, older HF patients is usually devoid the *Faecalibacterium* genus and enrichment of *Lactobacillus* species (Kamo et al., 2017).

#### Atrial fibrillation

Specific perturbations and disorder of gut microbesin fecal samples have been observed to accompany atrial fibrillation, one of the most prevalent and widespread arrhythmias. It has been reported that overgrowth of *Ruminococcu*s, *Streptococcus* and *Enterococcus*, together with reductions in *Faecalibacterium*, *Alistipes*, *Oscillibacter* and *Bilophila* occur in atrial fibrillation patients (Zuo et al., 2019a). The gut microtiota in both patients with persistent atrial fibrillation is significantly perturbed with elevated microbial diversity, distinct structures, and discrepant (Zuo et al., 2019b).

### Changes in blood microbial composition

Blood is traditionally considered to be a sterile environment devoid of any microorganisms under most healthy conditions. However, increasing evidence suggests the existence of blood microbiome. The blood microbiota of healthy humans is dominated by *Proteobacteria*, while the gut microbiota is predominantly composed of the bacterial phyla *Firmicutes* and *Bacteroidetes* (Castillo et al., 2019). The major route by which microbes enters the blood is thought to be through translocation from microbe-enriched organs (such as the gut) or via prenatal migration.

Circulating microbes may act as prime sources for microbial colonization of atherosclerotic plaques and subsequent development of inflammation and CVDs. CVD patients showed elevated concentrations of circulating microbes, with significantly elevated *Proteobacteria* and *Pseudomonadaceae* populations and decreased *Firmicutes Gammaproteobacteria*, *Bacillales* and *Staphylococcaceae* abundance (Dinakaran et al., 2014; Rajendhran et al., 2013). Increased levels of *Staphylococcus* spp. was found in the circulating blood in congenital heart disease (CHD), valvular heart disease (VHD), and ischemic heart disease (IHD) (Dinakaran et al., 2012).It has been reported that the cardiovascular outcomes of ST-segment elevation myocardial infarction (STEMI) patients are driven by intestinal microbe translocation into the systemic circulation. Increased abundances of *Lactobacillus*, *Bacteroides* and *Streptococcus* were detected in these STEMI patients, possibly as a result of tight junction disruptions in the gut barrier (Zhou et al., 2018). These changes in circulating microbes may induce chronic infection and inflammatory responses, leading to CVDs.

### Changes in microbial metabolites

**Figure 1:**
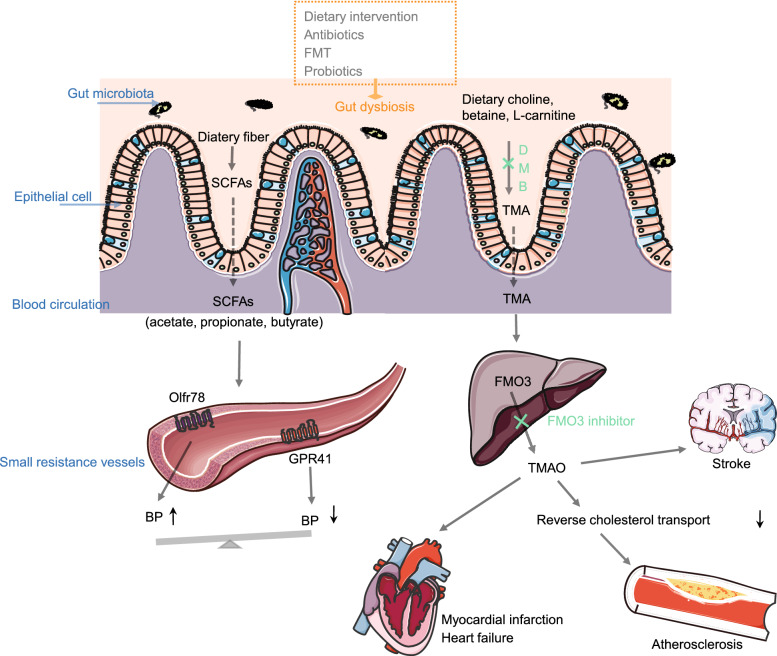
**Microbiota-associated metabolites involved into the pathogenesis of CVDs**. Nutrition can be metabolized to TMA by gut microbiota, which is inhibited by DMB. Most of TMA is absorbed into circulation and converted into TMAO by hepatic FMO3 (flavin monooxygenases). The circulaing TMAO may be indicators of MI, HF, peripheral artery disease, etc. Some intestianl microflora metabolite, such as SCFAs, can regulate blood pressure by combining with Olfr78 and GPR41. Microbiota-targeted therapeutics can alter the dysbiosis of gut microbiota

There are a number of intestinal microbial metabolites, such as vitamins, hormones, SCFAs, amino acid derivatives and antioxidants in the gut (Sekirov et al., 2010). These metabolites may be directly absorbed and enter the host circulation to migrate to distant organs. Alternatively, they maybe metabolized by host enzymes to serve as signaling molecules. The majority of microbe generated metabolites can exert synergistic effects in healthy individuals (Fig. 1).

#### *Short chain fatty acids* (*SCFA*)

Human gut is incapable of digesting complex carbohydrates in the form of dietary fiber for cell activities. However, the gut microbiota is able to utilize fibers through the fermentation process, leading to the production of SCFAs (Ahmad et al., 2019). SCFAs are saturated fatty acids that contain one to six carbon chains. Acetate, propionate and butyrate account for the majority of SCFAs in human body (Blacher et al., 2017). SCFAs serve as key regulators of anti-inflammatory responses as well as lipid metabolic pathways and gluconeogenesis. In addition, these molecules, in particular the butyrate, are viewed as energy substrates for epithelial cells of the intestines (Donohoe et al., 2011).

It is thought that SCFAs in the systemic circulation mediate the potential modulatory effects of the microbiota on CVDs. Numerous studies indicated that sodium butyrate and propionate produced by the gut microbiota are inversely correlated with the function of prorenin receptor-mediated intrarenal rennin-angiotensin system and contribute to lower blood pressure (Pluznick et al., 2013; Wang et al., 2017). Functionally, SCFAs absorbed into the bloodstream may mediate blood pressure effectors via the G protein-coupled receptors (GPCRs), such as Olfactory receptors 78 (Olfr78) and G protein-coupled receptor 41 (GPR41). Both receptors are localized in small resistance vessels, where they act divergently towards vascular tone. Under stimulation by SCFAs, GPR41 acts as a hypotensive protein to dilate resistance vessels in an endothelium-dependent manner. This hypotensive effect can be neutralized by Olfr78 (Natarajan et al., 2016). These two receptors provide the necessary functional balance to prevent excessive variation in blood pressures under healthy conditions (Pluznick, 2014). In addition, it has been reported that microbiota-derived SCFAs play an immunomodulatory role in oxidative stress attenuation and functional immune system maintenance. Dietary supplementation with 1% butyrate has been shown to slow atherosclerosis progression through its anti-inflammatory function and enhanced plaque stability (Aguilar et al., 2014). Moreover, SCFA propionate protects against cardiac hypertrophy, fibrosis, vascular dysfunction, and hypertension in a T cell-dependent manner (Bartolomaeus et al., 2019).

#### *Trimethylamine N-oxide* (*TMAO*)

Among the microbial metabolites, TMAO has gained considerable attention as a major contributing factor in CVDs. TMAO is a hepatic oxidation product of trimethylamine (TMA), which is primarily derived from bacterial metabolism of dietary choline and phosphatidylcholine (Wang et al., 2011). Studies on germ-free mice and human cohorts have indicated a positive linkage between plasma TMAO levels and increased CVD risk (Koeth et al., 2013; Tang et al., 2013; Tang et al., 2015; Wang et al., 2011; Wang et al., 2014). Mice fed with choline-rich and carnitine-rich diets showed elevated plasma TMAO levels and atherosclerotic plaque enhancement, while diet supplement depletion or intestinal microbiota suppression can eliminate TMAO generation and reduce atherosclerosis (Koeth et al., 2013; Wang et al., 2011). Thus, circulating TMAO may be a useful indicator for CVD diagnosis. Elevated TMAO levels may suggest increased risks for developing myocardial infarction (MI) (Li et al., 2017b), HF (Tang et al., 2014), peripheral artery disease (Senthong et al., 2016a), stroke (Tang et al., 2013; Wang et al., 2014) as well as stable coronary artery disease (Senthong et al., 2016b), independent of the traditional cardiac risk factors (Tang et al., 2013). Individuals taking broad-spectrum antibiotics result in depleted gut microbiota and show significant drops in TMAO levels (Tang et al., 2013; Wang et al., 2014). In addition, since diet is a major source of TMAO, individuals are suggested to avoid excessive consumption of foods rich in carnitine, choline and lecithin (Koeth et al., 2013) to reduce the risk of developing CVD.

Koeth et al. found that the presence of TMAO is associated with changes in plasma lipid, cholesterol and sterol metabolism (Koeth et al., 2013). *In vitro* and *in vivo* experimental evidences have revealed contributory mechanisms of TMAO on the vascular dysfunction, inflammatory responses and oxidative stress (Brunt et al., 2020; Seldin et al., 2016). The endoplasmic reticulum stress kinase PERK has been identified as a TMAO receptor (Chen et al., 2019). Interestingly, TMAO has been reported to play a protective role in hemodialysis patients with vascular injury, partly due to its inhibitory actions on AGE (Fukami et al., 2015).

Several choline analogues have been reported to reduce TMAO levels in the circulation. A natural compound 3,3-dimethyl-1-butanol (DMB), exists widely in vinegars, red wines and some grape seed oils, has been found to inhibit the microbial choline TMA lyase activity. This compound could inhibit atherosclerotic lesion development in Apoe^−/−^ mice without alterations in circulating cholesterol levels (Wang et al., 2015). Other choline analogues, such as fluromethylcholine (FMC), chloromethylcholine, bromomethylcholine, iodomethylcholine (IMC), are reported to be more potent TMA lyase inhibitors to lower plasma TMAO levels (Roberts et al., 2018).

#### Other metabolites

Phenylalanine (Phe), tryptophan (Trp) and tyrosine (Tyr) are aromatic amino acids that may influence immune, metabolic, and neuronal responses. Phe, an essential amino acid, is a metabolic precursor for Tyr, which maybe further processed into neurotransmitters, norepinephrine and adrenaline. Trp, also an essential amino acid, is a precursor for serotonin, another neurotransmitter. In patients with advanced atherosclerosis, the specific microbe-derived metabolite of Trp was significantly lower in plasma (Cason et al., 2018). A recent study showed that the gut microbiota metabolites of Tyr and Phe may be related to the severity of MI in rats (Lam et al., 2016). Further step into the mechanistic links between these aromatic amino acids metabolites and cardiovascular diseases is needed.

Phenylacetylglutamine (PAGln), which is produced as the dietary Phe converted into phenylacetic acid, has been reported to be associated with CVDs and major adverse cardiovascular events (Poesen et al., 2016). This gut microbiota-derived metabolite acts to enhance platelet activation-related phenotypes and thrombosis potential, signaling through adrenergic receptors (Nemet et al., 2020).

### Gaps in knowledge

Recent studies have highlighted the potential roles of microbiome dysbiosis in CVD diseases. Advances in genomic and metabolomics approaches have facilitated more in depth characterization and mechanistic exploration of these microbiome and their metabolites. However, most data are still making correlations and the exact molecular mechanisms underlying most observed phenomena remain unknown. Future studies focusing on microbe-microbe and microbe-host interactions may unravel how certain metabolic molecules function to modulate the disease process. It is also of great importance to understand more deeply on microbial pathways involved into the biosynthesis of CVD related metabolites, and the functional roles of these metabolites (Wang and Zhao, 2018). Results from these studies may provide essential scientific basis for the development of therapeutic interventions to prevent and treat CVD patients.

## Microbiota-targeted therapeutics

**Figure 2:**
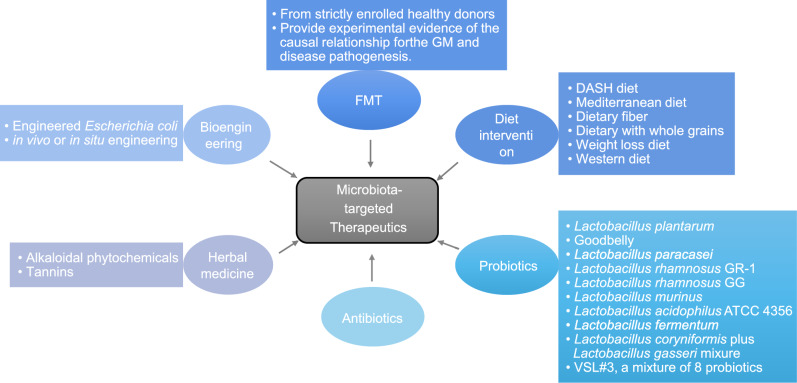
**Potential interventions associated with gut microbiota correction and CVDs improvement**. There are six strategies discussed in the present review, including dietary interventions, probiotics, antibiotics, FMT, bioengineering, and herbal medicine

Increasing evidence indicate that gut microbiota play an important role in the progression of CVDs. Thus, gut microbiota has emerged as an ideal target for disease prevention and treatment. Therapeutic strategies designed to manipulate gut microbiota composition and/or their metabolisms have been developed, including dietary intervention; probiotic, prebiotic, and antibiotic treatments; as well as fecal transplantation (Fig. 2). These strategies have improved blood pressure levels, normalized lipid profiles, and reduced body weight in some CVD patients.

### Dietary intervention

Dietary intervention has been widely practiced to alleviate chronic conditions. For example, heart-healthy diets rich in vegetables and high in fibers is thought to be beneficial to the cardiovascular system (De Filippis et al., 2016). Consumption of mediterranean diet and dietary approaches to stop hypertension (DASH) diet (rich in fruits, vegetables, legumes, and unsaturated fats with limited red meat intake) has been thought to reduce CVD incidence among participants with high cardiovascular risk (Appel et al., 1997).

It is widely accepted that dietary habits influence the composition and function of gut microbiota, thus modulate human health via digestion and absorption of various nutrients. These health effects can persist throughout an individual’s lifespan (David et al., 2014; Xu and Knight, 2015). For example, dietary fiber may alter the host microbiota compositions. Mice treated with excess mineralocorticoids and fed a high-fiber diet demonstrate increased abundance of the acetate-producing bacterium *Bacteroides acidifaciens*, down-regulation of a master cardiovascular regulator gene, and improved cardiovascular health and function (Marques et al., 2017). An intake of lower dietary fiber is associated with reduced microbial diversity and SCFA production but higher production of metabolites that are potentially detrimental to mucins. It is speculated that dietary fiber deprivation-induced degradation of the colonic mucus barrier is responsible for the enhanced susceptibility to infections (Desai et al., 2016). Mediterranean diet consumers exhibit reduced CVD incidence and elevated levels of fecal SCFAs-and fiber-degrading *Firmicutes* (Trichopoulou et al., 2009). A dietary scheme with more whole grains resulted in improvements in lipid profiles and blood pressure accompanied by concomitant reductions in opportunistic pathogens of the *Enterobacteriaceae* family and increases in gut barrier-protecting *Bifidobacteriaceae* bacteria (Xiao et al., 2014). A weight-loss diet consisting high intake of proteins but low fermentable carbohydrates improved the abundance of butyrate-producing *Roseburia* bacteria and *Eubacterium rectale* (Duncan et al., 2007). However, a Western diet rich in saturated fats and sugars and low in fiber is known to be associated with increased CVD risk. This diet reduces microbial diversity, decreases the levels of beneficial *Bifidobacterium* and *Eubacterium* bacterial population, increases the abundance of the mucin-degrading bacterium *Akkermansia* muciniphila, and promote T helper 17 (TH17) cell activity (Battson et al., 2018b; Wilck et al., 2017).

Diet interventions are relatively low cost and easily to administer for CVDs prevention and treatment. However, microbiome-based dietary approach may result in unintended consequences. For example, microbe-generated metabolites may act on multiple tissues and targets. The mechanisms remain unclear for most of the effects of dietary interventions (Turnbaugh, 2020). Due to the enormous heterogeneity in gut microbial composition and their functions, more personalized diets may be needed instead of a universal diet. In addition to food composition, cooking methods and the broad chemical diversity of plants further complicates the interplay between microbiota and CVDs.

### Antibiotics

Microbiota can be modulated with antibiotics and has been utilized to restore microbial communities and prevent CVDs. In one study, a 69-year patient with a long history (44 years) of hypertension and resistance hypertension showed lower blood pressure after combined antibiotic treatment (Qi et al., 2015). Separately, two months of dietary intervention with a broad-spectrum antibiotic cocktail reversed Western diet induced vascular dysfunction, which is a critical preclinical step in CVD progression (Battson et al., 2018a). Furthermore, vancomycin usage is associated with decreased myocardial infarct area (27% reduction) and improved post ischemic recovery of mechanical function (35%) along with effective reductions in the total numbers and groups of intestinal microbes (Lam et al., 2012).

Although antibiotics are commonly used against pathogens, they are non-discriminatory between pathogens and commensal microorganisms. Thus, antibiotic therapy may worsen the emergence of antibiotic-resistant bacteria. Consequently, consumption of antibiotics may lead to microbiota dysbiosis and anti-microbial resistance. In a meta-analysis of 33 studies including 20,779,963 participants, macrolide antibiotic administration was found to be associated with increased risk of sudden cardiac death (SCD) or ventricular tachyarrhythmia (VTA) (Cheng et al., 2015). Future studies are urgently needed to optimize the application of antibiotics in VCD-related patients.

### Probiotics

Probiotics contain beneficial bacteria intended to establish normal intestinal microbiome balance. One meta-analysis of clinical trials found that probiotic treatment may be effective in lowering total and low density lipoprotein (LDL) cholesterol levels, improving blood pressures, and in modulating inflammatory cytokines (Khalesi et al., 2014; Shimizu et al., 2015; Xue et al., 2017). *Bifidobacterium spp*. and *Lactobacillus spp.* are used widely and maybe the most promising probiotic species (Azad et al., 2018).

*Lactobacillus plantarum* is a probiotic supplement in the form of fermented plant foods (Molin, 2001). Rats fed with *Lactobacillus plantarum* showed reduced bacterial translocation, less mucosal inflammatory responses and increased abundances of the genera *Escherichia* and *Salmonella*. Dietary intake of *Lactobacillus plantarum* lowered serum levels of leptin and fibrinogen, two CVD risk factors, resulting in lower atherosclerosis in smokers (Naruszewicz et al., 2002). Among men with arteriosclerosis, consumption of drinks with high content of *Lactobacillus plantarum* increased the diversity of beneficial bacteria (Karlsson et al., 2010). Similarly, intake of Good belly (containing the strains *Lactobacillus plantarum* 299v and *Bifidobacterium lactis* Bi07) significantly reduced the infarct size and improve left ventricular function after MI (Lam et al., 2012).

*Lactobacillus paracasei* and *Lactobacillus rhamnosus* are enriched in dairy products (Molin, 2001). Consumption of *Lactobacillus rhamnosus* GR-1 contributes to the attenuation of left ventricular hypertrophy and improvement of systolic and diastolic left ventricular function after MI (Gan et al., 2014). Intervention with *Lactobacillus rhamnosus* GG can ameliorate atherosclerosis in a GM-associated manner (Chan et al., 2016a).

Probiotics with other *Lactobacillus* spp. also known to have positive effects on CVDs. *Lactobacillus murinus*-treated mice demonstrated resistance to high salt-induced hypertension due to the inhibition of TH17 cells (Wilck et al., 2017)*. Lactobacillus acidophilus ATCC* 4356 prevents atherosclerosis by inhibiting intestinal cholesterol absorption in ApoE(−/−) mice (Huang et al., 2014). Probiotic mixtures of *Lactobacillus* *fermentum*or, *Lactobacillu scoryniformis*, and *Lactobacillus gasseri* had cardiovascular protective effects in hypertensive rats. This is possibly due to improvements in vascular pro-oxidative and proinflammatory status. Changes in the cecal microbiota with increases in *Lactobacillus* *spp*. and decreases in *Bacteroides spp*. and *Clostridium spp*. may also contributed to the protective effects (Gomez-Guzman et al., 2015). VSL#3 is a mixture consists of 8 probiotics with anti-inflammatory potential. It has been shown that VSL#3 reduced the risk for atherosclerotic plaque rupture and vascular inflammation, as well as improved dyslipidemia profiles in overweight adults (Chan et al., 2016b; Lopez-Mejias et al., 2014; Mencarelli et al., 2012).

The benefits of relevant probiotics remain controversial. Animal model studies and human clinical trials revealed no beneficial effects of probiotics on CVDs (Fak and Backhed, 2012; Portugal et al., 2006; Vaghef-Mehrabany et al., 2017). These discrepancies may be partly due to differences in the probiotic strains or doses consumption, the experimental variation, and the heterogeneity of participants. As of today, most probiotic studies focused on populations with specific health issues, the general effect of probiotic consumption in healthy individuals requires further investigation (Khalesi et al., 2019).

### Fecal microbiota transplantation

Fecal microbiota transplantation (FMT) is a therapeutic strategy designed to transfer donor fecal samples into the gastrointestinal (GI) tract of a patient with a depleted microbiota, for the aim of restoring normal GI microbiota in patients. Depending on the source of the fecal microbiota for transplantation, FMT can be divided into allogenetic FMT (fecal material from a healthy donor) and autologous FMT (patients receive their own fecal material). Allogenic FMT are the most commonly used (Halkjaer et al., 2018). The fecal microbiota can be prepared in a lab for transplantation in four different ways: rough filtration, filtration plus centrifugation (FPC), microfiltration plus centrifugation (MPC), and purification. All these methods aim to minimize the processing time to minimize the loss of living bacteria (Zhang et al., 2018). FMT delivery routes include the upper gut, middle gut and lower gut. Microbiota capsules are delivered through the upper gut, while microbiota suspensions can be infused into the lower gut through colonic transendoscopic enteral tubing (TET) (Zhang et al., 2019).

The FMT approach has generated great interest when treating intestinal diseases, such as *Clostridium difficile* infection and inflammatory bowel diseases. Evidence suggests that FMT is markedly superior to antibiotic therapy in treating recurrent *Clostridium difficile* infection (van Nood et al., 2013). Recently, this strategy has also been successfully applied to patients with cardiometabolic disorders (Vrieze et al., 2012). Interestingly, obese patients with metabolic syndrome show enhanced insulin sensitivity when first infused with intestinal microbes from lean donors.

Successful FMT also provides solid evidence underlying the direct role of gut microbiota in disease pathogenesis. In those studies, fecal microbiota from healthy and pathological individuals was transplanted into germ-free (GF) rodents, and the recipient animals were analyzed for pathological changes (Maruvada et al., 2017). Majority of recipient rodents are reported to develop pathological phenotypes after fecal transplantation, however, some of them have commonly used small numbers of disease donors and conducted replication in larger numbers of individual mice (Walter et al., 2020). In addition, a substantial proportion of taxa in the human gut microbiota failed to colonize the recipient animals, suggesting unknown ecological factors may be absent in recipient rodents to facilitate human gut microbiota growth. Therefore, disease-associated alterations in humans are complex and difficult to replicate in animals such as rodents.

Despite the successful application of FMT in some patients, careful additional evaluations must be carried out before it can be widely implemented. First, FMT treatment alters levels of both harmful and beneficial bacteria, thus, adverse effects could be potentially induced into the recipient. Second, microbiome is diverse throughout the digestive tract, and it is important to obtain the most representative donor samples possible. Third, the potential transfer of endotoxins or infectious agents through FMT may lead to new GI complications. Fourth, a number of ethical issues further complicate FMT. In addition, informed consent, the availability of suitable healthy donors, safety and risk, remain to be carefully considered for all patients. Safe guard has to be established to monitor the FMT procedure and to protect both the patients and donors. These practices will promote further research into FMT and prevent abuse.

### Bioengineering

Bacteria are well suited to be genetically modified to have desired properties, such as a particular metabolic activity and produce a specific end product. Thus, it is possible to engineer the bacteria to possess more beneficial effects (Lam et al., 2019). Strains of *Escherichia coli* engineered to express particular genes that can alleviate rare metabolic diseases are now in clinical trials. For example, the SYNB1618 strain of *Escherichia coli* has been engineered to express proteins that are capable of degrading Phe, which is responsible for causing human phenylketonuria (PKU) disease (Isabella et al., 2018). To take one step further, *in vivo* and *in situ* engineering approaches, which are performed in the host rather than in the laboratory, have been described (Ronda et al., 2019).These genetic tools and the resulting microbial tools have ushered in an era of precision medicine treating the GM disorders.

### Herbal medicines

Herbal medicines are plant-derived materials that are mostly orally administered and are known to be effective in long-term clinical practice (Cheung, 2011). Although research on the effects of herbal medicines on the gut microbiota is still in its infancy, initial findings are encouraging the use of these medicines to help to treat gut microbiota-related illnesses.

Rhizoma coptidis (RC) is the dried rhizome of a medicinal plant from the family Ranunculaceae and contains alkaloids such as berberine, coptisine, and palmatine (He et al., 2016). Feeding of RC alkaloids to hyperlipidemic mice significantly changed the gut microbiota with increased abundance of *Akkermansia muciniphila*, *Sporobacter termitidis* and *Alcaligenes faecalis* while suppressing the abundances of *Parabacteroides distasonis* and *Escherichia coli*. Barbering-treated rats with high-fat diet (HFD)-induced hyperlipidemia exhibit reduced *Escherichia* populations and increased levels of beneficial bacteria (e.g., *Bacteroides* and *Blautia*) (Li et al., 2016). Alkaloid phytochemicals modulate both the gut microbiota and the bile acid pathway to reduce levels of triglycerides, total serum cholesterol, LDL cholesterol, lipopolysaccharide, and total bile acids in mice (Wu and Tan, 2019).

Persimmon tannin (PT) is a type of condensed tannin highly polymerized and non-absorbed in the intestine. It was reported to modify the intestinal bacterial microbiota composition by directly interacting with the colonic gut microbial ecosystem (Ozdal et al., 2016). The anti-hyperlipidemic and cholesterol-lowering effects of this compound on high-cholesterol diets fed SD rats was partly mediated by altered gut microbiota composition, such as increases in *Lactobacillus spp*. and *Bifidobacterium spp*. as well as reductions in *Escherichia coli* and *Enterococcus* populations (Zhu et al., 2018).

## Future perspectives

Increasing evidence suggest a link between the intestinal microbiome and CVD incidence. Studies have indicated that the microbiota interacts with the host through multiple pathways. Abnormal gut microbiota composition or microbial metabolites may be responsible for altering CVDs risks and its related pathological changes. Thus, novel therapeutic targets/strategies for preventing and treating CVD have been developed harnessing the potential use of gut microbiota.

Intense efforts are underway to advance the potential use of microbiota in CVD and other human diseases in general. First, identification of specific microbe strains rather than a general bacterial community is helpful to elucidate the contributions of specific microorganisms to disease progression. Second, future research may focus more on microbiome-mediated metabolites and their downstream functional consequences, while the current studies mostly explore the microbial composition. Third, personalized approaches for microbiota modification are urgently needed. This effort may be aided by microbiome profiling of individual patients for their metabolomic biomarker.
